# Role of mitochondria in doxorubicin-mediated cardiotoxicity: from molecular mechanisms to therapeutic strategies

**DOI:** 10.7150/ijms.94485

**Published:** 2024-03-11

**Authors:** Tianen Wang, Guoli Xing, Tong Fu, Yanchun Ma, Qi Wang, Shuxiang Zhang, Xing Chang, Ying Tong

**Affiliations:** 1 First Afliated Hospital, Heilongjiang University of Chinese Medicine, Harbin 150040, China.; 2 Brandeis University, Waltham, MA 02453, USA.; 3 Heilongjiang University of Chinese Medicine, Harbin 150040, China.; 4 Department of Cardiology, Guang'anmen Hospital, China Academy of Chinese Medical Sciences, Beijing, 100053, China.

**Keywords:** doxorubicin, cardiotoxicity, mitochondria, mitochondrial quality control

## Abstract

This comprehensive review delves into the pivotal role of mitochondria in doxorubicin-induced cardiotoxicity, a significant complication limiting the clinical use of this potent anthracycline chemotherapeutic agent. Doxorubicin, while effective against various malignancies, is associated with dose-dependent cardiotoxicity, potentially leading to irreversible cardiac damage. The review meticulously dissects the molecular mechanisms underpinning this cardiotoxicity, particularly focusing on mitochondrial dysfunction, a central player in this adverse effect. Central to the discussion is the concept of mitochondrial quality control (MQC), including mitochondrial dynamics (fusion/fission balance) and mitophagy. The review presents evidence linking aberrations in these processes to cardiotoxicity in doxorubicin-treated patients. It elucidates how doxorubicin disrupts mitochondrial dynamics, leading to an imbalance between mitochondrial fission and fusion, and impairs mitophagy, culminating in the accumulation of dysfunctional mitochondria and subsequent cardiac cell damage. Furthermore, the review explores emerging therapeutic strategies targeting mitochondrial dysfunction. It highlights the potential of modulating mitochondrial dynamics and enhancing mitophagy to mitigate doxorubicin-induced cardiac damage. These strategies include pharmacological interventions with mitochondrial fission inhibitors, fusion promoters, and agents that modulate mitophagy. The review underscores the promising results from preclinical studies while advocating for more extensive clinical trials to validate these approaches in human patients. In conclusion, this review offers valuable insights into the intricate relationship between mitochondrial dysfunction and doxorubicin-mediated cardiotoxicity. It underscores the need for continued research into targeted mitochondrial therapies as a means to improve the cardiac safety profile of doxorubicin, thereby enhancing the overall treatment outcomes for cancer patients.

## Introduction

Advances in drug development have enhanced significantly the survival of cancer patients. However, numerous chemotherapy drugs can induce adverse reactions, among which cardiovascular toxicity is the most prevalent and life-threatening. Anthracyclines are widely used chemotherapy drugs utilized in both adults and children for treating malignant tumors such as lymphoma, sarcoma, and breast cancer 1. The most frequently prescribed anthracycline drug is doxorubicin. Nevertheless, despite its effectiveness, up to a quarter of patients may develop doxorubicin-induced cardiotoxicity, which limits the utilization of this medication in clinical management 2, 3. Doxorubicin can induce cardiotoxicity through various mechanisms, including oxidative stress, induction of cell death via apoptosis or pyroptosis, abnormal intracellular calcium signaling, disrupted autophagy, metabolic disorder, endoplasmic reticulum stress, and mitochondrial structural/functional damage 4, 5. Elucidating the intrinsic correlations among these mechanisms is crucial for identifying therapeutic targets and designing strategies to reduce the side effects of doxorubicin on the cardiovascular system. Extensive research has focused on reducing intracellular oxidative stress to mitigate the occurrence of doxorubicin-mediated cardiotoxicity 6, 7. However, these efforts have largely failed 6, 8, indicating that additional mechanisms need to be addressed. It thus became clear that multiple and interdependent processes are involved in the cardiotoxic effects of doxorubicin.

The aim of this review is to summarize the pathological roles of mitochondrial dysfunction in the setting of doxorubicin-mediated cardiotoxicity. Since mitochondrial dynamics (determined by fission/fusion events), as well as mitophagy, are main targets of the mitochondrial quality control (MQC) program that sustains mitochondrial structure and function during organ and cellular stress 8, this review mainly discusses the molecular mechanisms underlying abnormal mitochondrial dynamics and disrupted mitophagy in cardiac cells exposed to doxorubicin. In addition, to provide insight and perspective for clinical management, we discuss potential therapeutics that aim to normalize mitochondrial dysfunction in patients with doxorubicin-mediated cardiotoxicity.

### Doxorubicin and its pharmacology

Doxorubicin is derived from the bacterium *Streptomyces peucetius* 9 and belongs to the anthracycline class of antibiotics 10. Chemically, it is composed of a naphthoquinone nucleus linked to a primary amine in its daunosamine sugar via a glycosidic bond, making it a cytotoxic drug. Pharmacologically, doxorubicin inhibits topoisomerase II 11, 12, thereby preventing DNA and RNA synthesis, which disrupts tumor proliferation and growth. Additionally, the anthracycline ring structure of doxorubicin intercalates into the DNA of human cells, covering the nucleotides 13. Within the anthracycline skeleton there is also a lipophilic, saturated hydroxyl-rich moiety adjacent to the amino sugar 14. Due to the hydrophilic nature of the amino sugar and the lipophilic properties of the napthoquinone core, doxorubicin can dissolve in both hydrophilic and lipophilic solvents. The lipophilic moiety of doxorubicin is acidic, while the hydrophilic amino sugar exhibits alkaline properties. In aqueous solution, maximum stability of the drug is observed at about pH 4.

The cytotoxic mechanism of doxorubicin on tumor cells is believed to be associated with its nucleotide base insertion and lipid binding activity 15. The insertion of doxorubicin hinders nucleotide replication and the activity of DNA and RNA polymerases 16. In addition, the interaction of doxorubicin with topoisomerase II forms a DNA-damaging complex, which further contributes to doxorubicin's tumor-killing actions 17. *In vitro* experiments have shown that proliferating cells treated with doxorubicin exhibit specific morphological changes and activate programmed cell death 17. As cancer is defined by uncontrolled cellular proliferation, doxorubicin has been found to effectively reduce tumor volume and size, thereby extending the survival of patients with cancer 17.

### Adverse effects of doxorubicin on cardiac muscle and heart function

The main limitation of doxorubicin use in clinical practice is its cardiotoxicity, as it has been widely reported to cause dose-dependent, progressive, and potentially fatal myocardial damage 18. Cardiotoxicity typically manifests within one year of treatment and is usually evident in the form of a decrease in left ventricular ejection fraction (LVEF) 19 20. It is worth noting that there are few studies reporting delayed cardiac toxicity in children years after doxorubicin treatment 21, 22. Doxorubicin-induced cardiotoxicity is primarily characterized by left ventricular dysfunction, dilated cardiomyopathy (DCM), and heart failure 23-25. Once it progresses to congestive heart failure, approximately 50% of patients die within two years 25. Numerous studies have confirmed the cumulative dose of anthracycline chemotherapy drugs, including doxorubicin, as a determining factor for the development of congestive heart failure. Mitry et al. reported that the risk of heart failure is 4% when the cumulative dose is below 500 mg/m^2^, but this risk increases to 36% when it exceeds 600 mg/m^2^ 26. In a retrospective study, the incidence of heart failure was 5% at a cumulative dose of 400 mg/m^2^, 16% at 500 mg/m^2^, 26% at 550 mg/m^2^, and rose to 48% when the dose reached 700 mg/m^2^ 19. A study by Lefrak et al. showed that the incidence of congestive heart failure was only 0.27% in patients receiving DOX treatment with a dose of no more than 550 mg/m^2^, compared to 30% in patients receiving doses exceeding 550 mg/m^2^ 27. At the molecular level, the cardiotoxic effects of doxorubicin include myocardial cell injury and apoptotic and necrotic cell death 28. Dexrazoxane is currently the only drug approved by the U.S. Food and Drug Administration (FDA) for the prevention of cardiotoxicity in anthracycline-based chemotherapy patients 29. However, its use is greatly limited by its association with reduced tumor response rates and potential risk of secondary malignancies.

### Role of mitochondrial dynamics in doxorubicin-induced cardiotoxicity

Mitochondria maintain their dynamic network through continuous fission and fusion reactions, which determine what is known as mitochondrial dynamics 30. Mitochondria undergo these processes to regulate their quantity, morphology, exchange of energy substrates, stability of genetic material, and conversion of extracellular signals to counteract external stress 31. Physiological mitochondrial fission mainly separates dysfunctional mitochondria from the healthy mitochondrial network, maintaining the stability of the mitochondrial population through the process of mitochondrial autophagy (i.e. mitophagy) 32. In contrast, pathological mitochondrial fission leads to a breakdown of the mitochondrial network and accumulation of fragmented mitochondria; this results in oxidative stress, via reactive oxygen species (ROS) overproduction, and impairs ATP synthesis, ultimately leading to cellular energy crisis 33. The regulation of mitochondrial fission is mainly controlled by the GTPase dynamin-related protein 1 (Drp1) 34. Drp1 translocates from the cytoplasm to the surface of mitochondria, where it firmly anchors to various Drp1 receptors 35. The receptors for Drp1 include mitochondrial fission 1 protein (Fis1), mitochondrial fission factor (Mff), and mitochondrial dynamics proteins of 49 and 51 kDa (Mid49 and Mid51) 33. Upon localization on the mitochondrial surface, Drp1 can assemble into multimeric ring-like structures around the mitochondrial surface, consuming GTP to constrict and divide the organelle to accomplish mitochondrial fission 35.

Mitochondrial fusion is the opposite process of mitochondrial fission, and proceeds via sequential merging of the outer and inner membranes of contiguous mitochondria 33. Physiological mitochondrial fusion fundamentally helps newly formed mitochondria to mature quickly, enhances the exchange and sharing of energy substrates, and reduces damage to mitochondrial genetic material 33. The fusion of mitochondrial membranes is regulated by two outer membrane fusion factors (mitofusin 1/2, Mfn1/2) and an inner membrane fusion factor (optic atrophy 1, Opa1). The outer membranes of adjacent mitochondria are tethered by dimeric structures involving Mfn1/2, followed by GTP consumption to promote outer membrane fusion. Although Opa1 has been shown to regulate inner membrane fusion in numerous studies, the specific mechanism is not yet fully understood 33.

Exposure to doxorubicin was shown to activate mitochondrial fission, evidenced by increased transcription of Drp1and mitochondrial fragmentation, in cardiomyocytes *in vitro* 36. Suggesting that mitochondrial fission contributes to doxorubicin-induced cardiomyocyte death, siRNA-mediated Drp1 knockdown was associated with a decrease in caspase-3 expression 36. Accordingly, Drp1-deficient mice were resistant to doxorubicin-mediated myocardial damage 36. Another study in a mouse model of doxorubicin-induced DCM confirmed that doxorubicin treatment triggered Drp1-related mitochondrial fission in cardiac cells, which subsequently activated the NLRP3 inflammasome and elicited pyroptosis 37. Nearly simultaneously, Liang et al. demonstrated that phosphorylation at Ser616 triggered Drp1-dependent unchecked mitochondrial fission, leading to cytochrome c leakage and cardiomyocyte death in mice treated with doxorubicin 38. Several studies reported also the important role played by Drp1 receptors in mitochondrial fission during doxorubicin-mediated myocardial damage. Zhou et al. showed that doxorubicin administration suppresses the expression of Foxo3a, a transcriptional inhibitor of Mid49, in the mouse heart and in cultured cardiomyocytes, resulting in Mdi49 upregulation and increased mitochondrial fission. Accordingly, cardiac-specific Foxo3a transgenic mice and cardiomyocytes transfected with Mid49 siRNA showed resistance to doxorubicin-mediated toxicity 39.

Interestingly, the stimulatory effect of doxorubicin on mitochondrial fission was reported to be compounded by negative regulation of the expression of key markers of mitochondrial fusion, namely Mfn1/2 and Opa1, in heart tissue 40. In addition to these changes in protein expression, mass spectrometry and mutagenesis analysis showed that doxorubicin induces Opa1 acetylation at Lys926/931 residues, possibly due to reduced Sirt3 expression 41. By comparison, either Sirt3 overexpression or transfection of a deacetylation-mimetic version of Opa1 was able to normalize mitochondrial morphology and preserve mitochondrial networking, attenuating cardiomyocyte death in the presence of doxorubicin 41. Similarly, administration of ethanolic extracts of *Rhaponticum uniflorum* (L.) DC inflorescence (loulu flowers) to H9c2 cardiomyocytes upregulated Mfn1 and Opa1 expression, prevented doxorubicin-mediated NF-κB activation, and reduced mitochondria-dependent apoptosis 42. Notably, a study in mice suggested that physical exercise may be an effective way to balance mitochondrial fission and fusion, prevent mitochondrial dysfunction, and neutralize oxidative damage in cardiac tissue following doxorubicin exposure 43.

### Mitophagy

Mitophagy is a protective process by which damaged mitochondria are degraded to prevent the accumulation of non-functional mitochondrial fragments 33. Moreover, through mitophagy, mitochondrial membranes and internal macromolecules are broken down into amino acids, glucose, and nucleotides, used in cellular energy, metabolic, and biosynthetic reactions 44. Mitophagy is broadly classified into two types, i.e. receptor-dependent and receptor-independent. The first is mainly mediated by specific proteins on the surface of mitochondria 45, and results in the formation of mitophagosomes. Receptor-independent mitophagy is mainly mediated by Parkin 46, which promotes the ubiquitination of mitochondrial proteins and fusion of mitochondria with lysosomes 33. Mitochondrial surface proteins involved in mitophagy include BCL2/adenovirus E1B 19 kDa protein-interacting protein 3 (Bnip3) 47, FUN14 domain-containing protein 1 (Fundc1) 48, and Nix 33.

Assays employing the novel dual-fluorescent mitophagy reporter mt-Rosella showed that doxorubicin exposure stimulates mitophagy in cardiomyocytes 36. Interestingly, deletion of Drp1 significantly suppressed mitophagy activation, suggesting that Drp1-dependent mitochondrial fission stimulates mitophagy in the setting of doxorubicin-mediated myocardial dysfunction. Highlighting the central role of mitophagy in this clinical condition, deletion and overexpression of Parkin in doxorubicin-treated cardiomyocytes were linked to decreased and increased cell death, respectively 36. In accordance with these findings, a study found that inhibition of mitophagy using liensinine, a plant-derived alkaloid, attenuated doxorubicin-caused cardiotoxicity by inhibiting cardiomyocyte apoptosis 38. Similarly, a recent study in a rat model of doxorubicin-induced chronic heart failure demonstrated that application of moxibustion, a therapy used in traditional Chinese medicine (TCM), to certain acupressure points had cardioprotective effects related to mitophagy inhibition. The latter was evidenced by decreased LC3II/LC3I ratio, attenuated Fundc1 expression, and increased p62 levels in heart tissue 49. However, as reviewed by Wallace et al. 50, some studies suggested that doxorubicin actually halts autophagic fluxes in cardiac cells, pointing to inhibition of lysosome acidification and subsequent accumulation of autophagosomes and autolysosomes as important determinants of DOX-induced cardiotoxicity.

In fact, a study by Hull et al. suggested that the activation and inactivation of Parkin/PINK1-mediated mitophagy may be related to the duration of doxorubicin treatment 51. Specifically, these authors reported that Parkin/PINK1 protein expression levels show a biphasic pattern in the hearts of mice treated with doxorubicin: initially (up to day 8 post-treatment), a significant decrease in Parkin/PINK1 expression levels, indicative of mitophagy suppression, was observed; however, by day 14 post-treatment there was a significant increase in Parkin/PINK1 expression, suggesting enhanced mitophagy. Moreover, coincident with delayed mitophagy stimulation, upregulated cardiac expression of the mitochondrial fission-related protein Fis1 was recorded 14 days after doxorubicin treatment 51. These results suggest that early suppression of mitophagy contributes to the cardiotoxic effects of doxorubicin, by determining late and ineffective removal of damaged mitochondria.

Meanwhile, there is evidence that doxorubicin administration is associated with over-activation of mitophagy, albeit with concomitant disruption of lysosomal function and interrupted mitophagy completion 33. Thus, it is believed that upon doxorubicin exposure, acute inhibition of mitophagy in heart tissue contributes to the accumulation of dysfunctional mitochondria, which promotes oxidative damage and cardiomyocyte apoptosis. In turn, late and chronic activation of mitophagy would lead to depletion of the mitochondrial population, which drastically lowers ATP synthesis and triggers myocardial dysfunction 52.

### Mitochondria-targeted therapies for doxorubicin-induced cardiotoxicity

In light of the essential roles of mitochondrial dynamics and mitophagy in regulating mitochondrial homeostasis, several investigations sought to evaluate the cardioprotective effect of pharmacological targeting of mitochondrial dynamics/mitophagy on doxorubicin-induced myocardial dysfunction (Table 1). Mdivi-1 is a mitochondrial fission inhibitor that interrupts the interaction between Drp1 and its receptors. Supplementation of Mdivi-1 was shown to prevent doxorubicin-induced mitochondrial fission and consequently reduce apoptosis in rat H9c2 53 and human AC16 54 cardiomyocytes.

Likewise, a study in rats revealed that Mdivi-1 treatment significantly attenuated doxorubicin-induced mitochondrial dysfunction, decreased cardiac inflammation, reduced cardiomyocyte apoptosis, and improved heart function 53. Although the beneficial effects of Mdivi-1 against mitochondrial stressors are broadly confirmed by cellular and animal studies, toxicity concerns significantly limit its clinical utilization. Therefore, and in view of Mdivi-1 pre-clinical success, development of safer mitochondrial fission inhibitors is of great interest in cardiovascular medicine.

Oseltamivir is an inhibitor of influenza virus neuraminidase, widely used for the treatment of flu. Treatment with oseltamivir is reported to repress Drp1-mediated mitochondrial division and thus reduce heart dysfunction in doxorubicin-treated rats 55. Similarly, Shenmai injection, a TCM formulation based on *Panax ginseng* and *Ophiopogon japonicus* extracts, was shown to not only inhibit Drp1-related mitochondrial fission but also promote Opa1-induced mitochondrial fusion, hence reducing doxorubicin-mediated cardiomyocyte death 56.

Melatonin is an endogenous hormone that acts a s a central regulator of the circadian rhythm. Several studies have reported that melatonin is also a powerful antioxidant agent. Incubation with melatonin effectively reduced doxorubicin-induced mitochondrial fragmentation and thus maintained viability in cardiomyocytes *in vitro* 57. Importantly, in doxorubicin-injected rats, melatonin supplementation greatly enhanced mitochondrial fusion through upregulation of Mfn1/2 expression 57. These results suggested that melatonin regulates mitochondrial dynamics through restricting mitochondrial fission and restoring mitochondrial fusion. Considering its endogenous and non-toxic nature, exogenous supplementation of melatonin may prove to be beneficial for cancer patients receiving doxorubicin therapy.

Sacubitril/valsartan is a novel inhibitor of the rennin-angiotensin system (RAS) which is now widely prescribed for patients with heart failure. Treatment with LCZ696, a component of sacubitril/valsartan, was found to reduce Drp1 activation and decrease cardiomyocyte death in mice with doxorubicin-induced DCM 58. Due to its recognized cardioprotective actions, especially prevention of progression of heart failure, the use of sacubitril/valsartan during doxorubicin therapy may be a promising strategy to prevent or attenuate myocardial dysfunction.

Supplementation with vitamin D, an essential micronutrient with high antioxidant properties, was shown to not only reduce oxidative stress but also alleviate Drp1-dependent mitochondrial fission in a mouse model of doxorubicin-induced cardiomyopathy 7. However, human studies are required to further validate the efficacy of Vitamin D in treating this condition.

Luteolin, a widely occurring flavonoid found in various plant sources such as broccoli, pepper, thyme, and celery, has been extensively studied for its neuroprotective effects. Luteolin exhibits antioxidant properties and possesses immunomodulatory activity in both *in vitro* and *in vivo* settings, making it a promising compound for therapeutic applications for doxorubicin-elicited cardiomyopathy. In doxorubicin-treated H9C2 cells, incubation with luteolin relieved mitochondrial fission, improved mitochondrial performance, and attenuated apoptosis 59.

Paeonol is a natural antioxidant derived from the root bark of *Paeonia suffruticosa* that is approved in China to alleviate pain and treat inflammatory conditions. Administration of paeonol was found to reverse Mfn2-related mitochondrial fusion in a manner depending on increased Stat3 expression, improving mitochondrial function and cardiac behavior in rats with doxorubicin-induced cardiomyopathy 60. Given the important role played by Sirt3 in regulating the activity of mitochondrial fusion, the use of Sirt3 agonists has potential therapeutic value in this medical condition. Administration of honokiol (HKL), a natural extract of the bark of the magnolia tree that acts as a Sirt3 activator, enhanced Sirt3 activity and reduced mitochondrial damage and cell death in rat neonatal cardiomyocytes by inhibiting ROS production and stimulating Mfn1/Opa1-mediated mitochondrial fusion. Moreover, these mechanisms accounted for effective cardioprotection in rats exposed to doxorubicin 61. Similarly, HKL treatment mitigated doxorubicin-induced cardiotoxicity in mice by suppressing cardiac inflammation and oxidative stress, resulting in improved cardiac function and reduced cardiomyocyte apoptosis 62.

Treatment with total flavonoids derived from *Selaginella tamariscina* (P.Beauv.) Spring (TFST) was found to attenuate doxorubicin-induced cardiotoxicity in mice 63. This effect was correlated with enhanced Mfn2-mediated mitochondrial fusion, reduced mitochondrial dysfunction, as well as Mfn2/PERK pathway activation leading to inhibition of endoplasmic reticulum stress 63.

Considering the deleterious effects on heart function of exacerbated mitophagy during long-term doxorubicin treatment, pre-clinical research has also addressed the therapeutic potential of selective/specific inhibitors of mitophagy. Acetylcholine receptors (AChRs) were proposed to participate in the regulation of mitophagy, and experimental attempts have been made to evaluate whether activation of AChRs correlates with mitophagy inhibition. In a mouse model of doxorubicin-induced cardiomyopathy, administration of PNU-282987 or bethanechol, two AChR agonists acting respectively on 7nAChR and mAChR, was shown to confer cardioprotection related to prevention of abnormal mitophagy activation, improved ATP synthesis, and enhanced cardiomyocyte survival 64.

Ellagic acid, a natural polyphenolic compound abundant in fruits and vegetables, shows potential as a therapeutic agent to modulate mitophagy. In a mouse model of doxorubicin-induced cardiomyopathy, treatment with ellagic acid reduced the levels of mitochondria-associated Bnip3, resulting in attenuation of both mitophagy and cardiomyocyte death. Moreover, ellagic acid suppressed also mitochondrial injury and cell death in Bnip3-overexpressing or hypoxic cardiomyocytes *in vitro* 65.

In their review 66, Cocetta et al. elucidate resveratrol's potential as a chemosensitizer in cancer treatment. This polyphenolic compound, ubiquitous in various plants, has been shown to interact with conventional chemotherapeutics, spanning alkylating agents to mitotic inhibitors, enhancing their efficacy through modulation of cancer pathways. This work illuminates the promise of resveratrol in augmenting therapeutic outcomes and underscores the imperative for further clinical inquiry to address its bioavailability challenges. Notably, resveratrol emerges as a viable candidate for mitigating doxorubicin-induced cardiac events, heralding a significant stride in oncological adjunct therapy.

Quagliariello et al. delve into the creation of nano-encapsulated Coenzyme Q10 (CoQ10) formulations, aimed at bolstering cardioprotective and hepatoprotective responses against anthracycline-induced toxicities, notably from doxorubicin and trastuzumab 67. This research 67 addresses the critical issue of anthracyclines triggering adverse cardiac and hepatic outcomes, proposing an innovative nano-emulsion technique to enhance CoQ10 delivery. Their findings reveal that such nano-encapsulated CoQ10 formulations significantly reduce oxidative stress and inflammation, suggesting a promising avenue to improve cancer therapy patient outcomes by minimizing cardiotoxic and hepatotoxic side effects 67.

## Outlook and conclusion

Doxorubicin has broad-spectrum anti-tumor effects and is widely used in clinical practice to improve the prognosis of cancer patients. However, its use is restricted by cardiotoxicity associated with cumulative dose-dependent mitochondrial damage, evidenced by dysregulated mitochondrial dynamics, disruption of mitochondrial fusion, and mitophagy imbalance. Disruption of mitochondrial structure and function triggers cellular oxidative stress, intracellular calcium overload, endoplasmic reticulum stress, and damages enzymes, lipids, and nucleic acids in cardiac cells.

Although the roles of abnormal mitochondrial dynamics and disrupted mitophagy in doxorubicin-induced cardiotoxicity have been extensively studied, effective preventive strategies and clinically applicable cardioprotective drugs are limited. Some studies have explored the cardioprotective effects of small molecule compounds that target mitochondrial dynamics and mitophagy. However, these compounds have limited evidence of safety and efficacy for clinical translation. Likewise, although promising results have been reported using natural plant and animal extracts, as well as chemically synthesized drugs, their efficacies have been so far mostly assessed only in animal and cellular studies. The characteristics of cancer patients are admittedly far more complex than those of laboratory animals. In cancer patients, doxorubicin-mediated cardiotoxicity may be more pronounced and severe due to advanced age and pre-existing organ damage. Therefore, clinical data are needed to assess the effectiveness of drug interventions in preventing doxorubicin-induced cardiotoxicity. Likewise, further research on the molecular actions of anthracycline derivatives is needed to reduce off-target toxicity. Developing safer and more tumor-targeted doxorubicin analogues that may be used in combination with cardioprotective therapy will help curtail the incidence of doxorubicin-induced cardiotoxicity in cancer treatment.

## Figures and Tables

**Table 1:** Mitochondria-targeted therapies for doxorubicin-induced cardiotoxicity

Drug	Model	Effects on mitochondrial dynamics/mitophagy	Cardiac phenotype	References
Mdivi-1	Rats	Inhibition of mitochondrial fission	Decreased oxidative stress, inflammation, and myocardial injury	53
Mdivi-1	Human AC16 cells	Inhibition of mitochondrial fission	Increased mitochondrial membrane potential, mtDNA copy number, and cardiomyocyte survival	54
Oseltamivir	Rats	Inhibition of mitochondrial fission and Parkin-related mitophagy	Decreased oxidative stress, reduced cardiomyocyte apoptosis	55
Shenmai injection	Mice	Inhibition of mitochondrial fission and activation of mitochondrial fusion	Improved mitochondrial respiratory function, attenuated oxidative stress, and decreased cardiomyocyte apoptosis	56
Melatonin	Rats	Inhibition of mitochondrial fission and activation of mitochondrial fusion	Improved mitochondrial metabolism, attenuated cardiomyocyte death	57
LCZ696	Mice	Inhibition of mitochondrial fission	Increased mitochondrial respiration complex I activity and ATP production, attenuated cardiomyocyte apoptosis	58
Vitamin D	Mice	Inhibition of mitochondrial fission	Decreased ROS production and attenuated mitochondrial damage	7
Luteolin	Zebrafish and mice	Inhibition of mitochondrial fission	Decreased cardiomyocyte death, preserved ventricular function	59.
Paeonol	Rats	Activation of mitochondrial fusion	Decreased mitochondrial oxidative stress and cardiomyocyte apoptosis, improved heart function	60
Honokiol	Mice	Activation of mitochondrial fusion	Reduced oxidative stress and cardiomyocyte death, improved heart function	61
Total flavonoids of *Selaginella tamariscina* Spring	Mice	Activation of mitochondrial fusion	Decreased levels of myocardial injury markers, attenuated oxidative stress, preserved mitochondrial potential, increased cardiomyocyte survival	63
Ellagic acid	Rats	Inhibition of mitophagy and mitochondrial fission	Decreased mPTP opening, preserved mitochondrial membrane potential, reduced cardiomyocyte death	65
